# Anthropogenic impacts on mosquito populations in North America over the past century

**DOI:** 10.1038/ncomms13604

**Published:** 2016-12-06

**Authors:** Ilia Rochlin, Ary Faraji, Dominick V. Ninivaggi, Christopher M. Barker, A. Marm Kilpatrick

**Affiliations:** 1 Suffolk County Vector Control, 335 Yaphank Avenue, Yaphank, New York 11980, USA; 2 Center for Vector Biology, Rutgers University, 180 Jones Avenue, New Brunswick, New Jersey 08901, USA; 3 Salt Lake City Mosquito Abatement District, 2020 North Redwood Road, Salt Lake City, Utah 84116, USA; 4 Department of Pathology, Microbiology and Immunology, School of Veterinary Medicine, University of California, Davis, 1 Shields Avenue, Davis, California 95616, USA; 5 Department of Ecology and Evolutionary Biology, 1156 High Street, University of California, Santa Cruz, Santa Cruz, California 95064, USA

## Abstract

The recent emergence and spread of vector-borne viruses including Zika, chikungunya and dengue has raised concerns that climate change may cause mosquito vectors of these diseases to expand into more temperate regions. However, the long-term impact of other anthropogenic factors on mosquito abundance and distributions is less studied. Here, we show that anthropogenic chemical use (DDT; dichlorodiphenyltrichloroethane) and increasing urbanization were the strongest drivers of changes in mosquito populations over the last eight decades in areas on both coasts of North America. Mosquito populations have increased as much as tenfold, and mosquito communities have become two- to fourfold richer over the last five decades. These increases are correlated with the decay in residual environmental DDT concentrations and growing human populations, but not with temperature. These results illustrate the far-reaching impacts of multiple anthropogenic disturbances on animal communities and suggest that interactions between land use and chemical use may have unforeseen consequences on ecosystems.

## Abstract (web summary)

Insect populations have fluctuated enormously over the past century, with many changes being attributed to anthropogenic climate change. Rochlin *et al*. show that the pesticide DDT and increasing urbanization are more strongly associated with changes in the number and diversity of mosquitoes on both coasts of North America.

Many animal species have shifted their geographic distributions over the past four decades, with the majority moving or expanding poleward or upward in elevation^1^,^2^,^3^,^4^. Changes in distributions often include more tropical species invading temperate habitats, and more temperate species disappearing from sites as habitats become warmer^2^. These shifts have been especially apparent in insects and other arthropods, possibly because of their high reproductive rate and mobility^1^,^5^,^6^,^7^. Changes in geographic ranges of arthropods are of public health significance, because distributional shifts of insect and tick vectors could alter and expand the range of diseases such as dengue, Zika, chikungunya, malaria and Lyme^8^,^9^,^10^.

Many recent latitudinal range changes of insects and diseases have been correlated with increased temperatures caused by a rise in global CO_2_ and other greenhouse gases^3^,^4^,^10^. Perhaps as a result, climate change has been predicted to increase or shift the distribution and incidence of many vector-borne diseases^11^,^12^. However, despite numerous predictions of shifts in the distribution of vector-borne diseases with climate change, there have been no analyses of the links between climate and long-term variation in mosquito or tick populations that include continuous datasets pre-dating the 1960s^13^.

Two additional large-scale environmental impacts that may have influenced insect populations over the past century are land use change, including urbanization, and the application of organochlorine pesticides (mostly DDT) in agriculture, forestry and public health. More than 2.8 billion kg of DDT was deposited worldwide, including 600 million kg in the US from the 1940s through the 1970s^14^,^15^. Although Europe and the US, the two regions where the majority of data on long-term insect population fluctuations have originated^3^,^6^,^7^,^16^, were subjected to especially heavy DDT loads, DDT use was global in scope^15^,^17^,^18^,^19^. The most severe environmental impact was on terrestrial and aquatic invertebrate fauna^17^,^18^. DDT was unlike any insecticide used before or after this period. Drastic reductions in the abundance of many insect orders including Ephemeroptera, Lepidoptera and Diptera, persisted for as long as 12–18 months in terrestrial and aquatic ecosystems following a single high-dose DDT application^18^,^20^. Birds of prey were also impacted by DDT through their diet, including declines in abundance of osprey (*Pandion haliaetus*), bald eagles (*Haliaeetus leucocephalus*) and other raptors in the 1950s through 1970s^18^,^21^. Despite the well-known devastating effects of DDT use on insect communities, most previous analyses of insect abundance and distribution have examined only temperature as a possible driver^1^,^3^,^5^,^7^,^16^. Thus, changes in abundance or distribution that have been attributed solely to climate change in previous studies may have been caused, wholly or in part, by other factors^6^.

We analyzed climate (temperature and precipitation), land use (urbanization) and DDT use and concentration in the environment (from sediment cores) as drivers of mosquito abundance and community composition using previously unpublished eight-decade-long datasets from New York (NY, 1938–2012) and New Jersey (NJ, 1932–2012) on the east coast of North America and a six-decade long dataset from the west coast in California (CA, 1954–2006), all collected with a consistent trapping methodology^22^. Urbanization was the dominant land use change in the study regions^23^, and we used a surrogate measure of urbanization, human population size, because available land use data do not cover the eight-decade temporal span of the mosquito data. The mosquito communities in these regions include vectors of many pathogens already established in North America, including West Nile virus, eastern equine encephalitis virus and St Louis encephalitis virus, and also potential vectors for pathogens that have recently been or are likely to be introduced in the near future, including Zika, chikungunya and Rift Valley fever viruses^24^,^25^,^26^.

We performed two sets of analyses. First, we examined trends in mosquitoes in the three study regions that grouped all species together. Next, we determined whether temperature effects could be masked by differential responses of different mosquito species to warming. To examine this possibility, we performed additional analyses on subsets of mosquito species grouped based on their geographical ranges in eastern United Sates. In both sets of analyses, we found that changes in mosquito communities were strongly correlated with changes in DDT concentration increases and urbanization, but were mostly uncorrelated with changes in annual temperature. The effects of land and chemical use on animal communities may exceed that of climate change.

## Results

### DDT and land use influence mosquito populations

**Figure 1:**
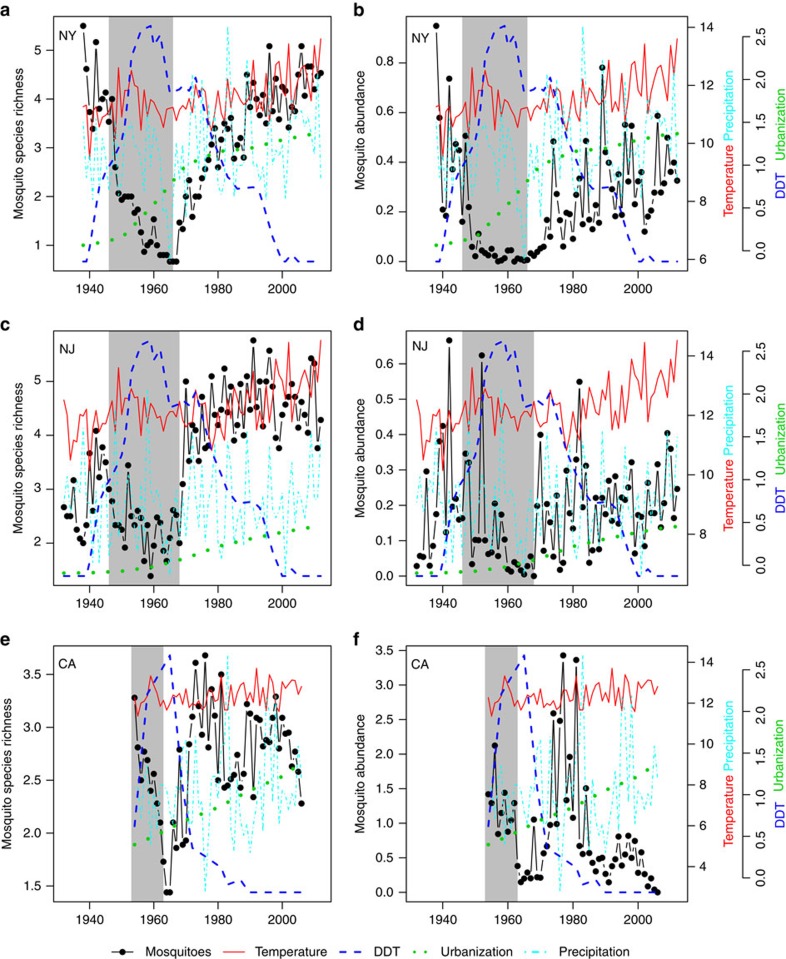
Time series of species richness and abundance of mosquitoes. Left panels and left axis show mosquito species richness, expressed as the average number of species collected per trap per night from New York (NY) (**a**), New Jersey (NJ) (**c**), and California (CA) (**e**). Right panels show mosquito relative abundance per trap night expressed as *Z*-scores from New York (NY) (**b**), New Jersey (NJ) (**d**) and California (CA) (**f**). For all panels, the first right axis shows temperature and precipitation. Temperature (solid red line) is the yearly average in degrees Celsius calculated from November of the preceding year through the following October. Precipitation (dashed light blue line) is the yearly average in centimeters. The second right-hand *y* axis shows DDT usage and urbanization, again for all panels. Relative DDT concentration (dashed dark blue line) is expressed as a *Z*-score and the period of DDT use by the mosquito control districts is indicated by the grey rectangle. Urbanization (dotted green line) is expressed as the human population in millions in NY and NJ and in 100,000s in CA.

**Figure 2:**
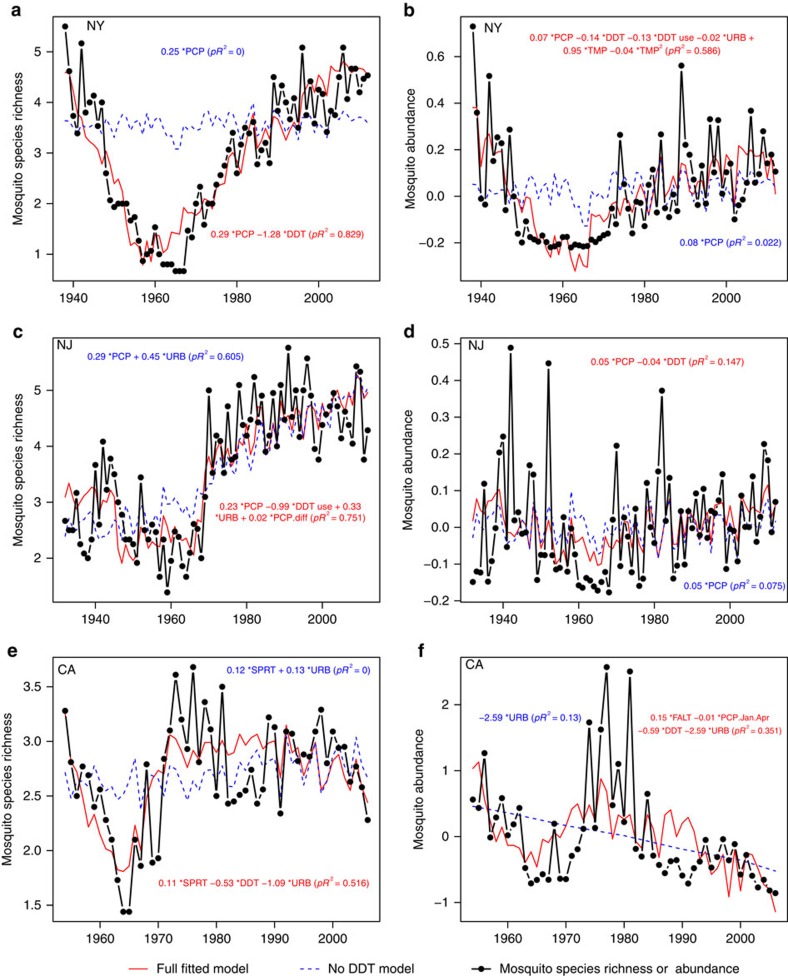
Fitted models and observed data of mosquito species richness and abundance. Left panels and left axis show mosquito species richness, expressed as the average number of species collected per trap per night from New York (NY) (**a**), New Jersey (NJ) (**c**), and California (CA) (**e**). Right panels show mosquito relative abundance per trap night expressed as *Z*-scores from New York (NY) (**b**), New Jersey (NJ) (**d**), and California (CA) (**f**). The equations in each panel give the fitted regression model with all significant predictors (*P*<0.05) and fraction of variance explained pseudo-*R*^2^ (*pR*^2^) from the generalized least squares models. Full model is shown in red, whereas the reduced model excluding DDT parameters is shown in blue (dashed line). The abbreviations designate DDT concentration (DDT, *Z*-scores), DDT use by the mosquito control districts (DDT use, yes/no), precipitation (PCP, standardized precipitation index), total precipitation in January through April (PCP.Jan.Apr), precipitation difference [April+March]−[January+February] (PCP.diff), urbanization (URB, human population in 100,000), spring (SPRT) and fall (FALT) temperatures. For relative importance of the variables in the model see Table 1.

There were enormous changes in mosquito communities over the past century, with species richness and abundance increasing two to tenfold over the past five decades (Fig. 1; all correlations with year were positive and significant (in Pearson's correlations, all *P*<0.02), except mosquito abundance in CA). Although there were significant increases in temperature over this period, multiple regression analyses suggested that long-term trends in mosquito communities were driven primarily by variation in DDT use and persistence and long-term increases in urbanization, with some of the year-to-year variation driven in part by precipitation, which showed little long-term trend (Figs 1 and 2; Table 1). DDT effects were consistently negative on both mosquito abundance and species richness, whereas the effects of urbanization were more variable, with significant negative effects on abundance in New York and California, and significant positive effects on mosquito species richness in New Jersey and California.

### Temporal trends in mosquito populations and DDT

Across all three datasets, mosquito species richness and abundance decreased, often precipitously, during the period of DDT use and then increased afterward, as the concentration of DDT in the environment decreased (Fig. 1). In NY the recovery was slow and it took mosquito communities nearly 40 years to reach pre-DDT levels. In CA and NJ recovery was much faster, and in NJ mosquito species richness continued to increase above pre-DDT levels. In CA mosquito richness recovered as soon as DDT concentrations declined and remained at pre-DDT levels, whereas abundance showed an initial spike after DDT concentrations waned, but then declined to much lower levels. In summary, while patterns of DDT use and concentration were sufficient to explain most of the long-term trends in NY, the data and analyses from NJ and CA indicate that long-term increases in urbanization were also important (Figs 1 and 2; Table 1).

### Lack of correlations with temperature

**Figure 3:**
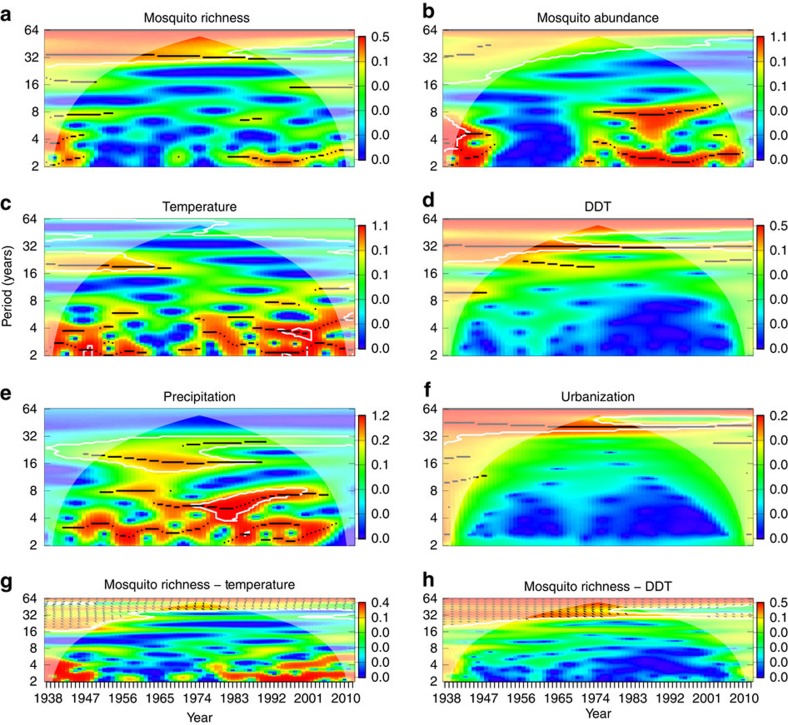
Wavelet analysis of time series. (**a**–**f**) The power level of each time series (mosquito species richness (**a**), mosquito abundance (**b**), temperature (**c**), DDT (**d**), precipitation (**e**) and urbanization (**f**)) from New York using a Morelet wavelet at each Fourier period or frequency (*y* axis in years) over the length of the time series (*x* axis) with warmer colours indicating higher power levels (right legends). Black lines indicate power ridges. The whitish cone indicates parts of the analysis influenced by edge-effects. The bottom two panels show the cross-wavelet coherence spectrum for two pairs of time series ((**g**) mosquito richness—DDT and (**h**) mosquito richness—temperature), with white outlines surrounding significant coherence correlations (*P*<0.1) and arrows indicating phase differences (arrows to the right indicate in-phase). The bottom two panels indicate moderate (higher periods) or non-significant (lower periods early and late in the time series) coherence between mosquito richness and temperature across most frequencies and parts of the time series and strong coherence between DDT concentration and mosquito richness across most of the time series at frequencies of 32 and 64 years.

Surprisingly, despite increases during the last five decades, annual average temperature was non-significant in most analyses for all three regions, and very weak in the single analysis in which it was significant, and temperature was never significant without DDT in the model (Table 1). In the single significant relationship (mosquito abundance in New York), the fitted model indicated that mosquito abundance varied by 5% across the range of annual temperatures in a hump-shaped pattern with a peak at 12 °C (Table 1). Wavelet analyses suggested the absence of temperature effects may be due, in part, to the differences in the frequency of temporal variation, which was primarily decadal (∼32 year period) for mosquito species richness and 3 and 8 year periods for mosquito abundance, whereas variation occurred primarily over 2–3 year and 20 year periods for temperature (Fig. 3). There was little evidence of non-linearity in the effects of temperature on mosquito richness or abundance; quadratic or other nonlinear transformations of temperature were non-significant (in regression analyses, all *P*>0.1) in all but the one analysis of mosquito abundance and richness (Fig. 2; Table 1). We also examined seven other temperature variables, and only the dataset from California suggested that temperature might be influencing either abundance or species richness. Temperature predictors were on the edge of significance and weaker than other predictors such as DDT contributing only about 5% to the model goodness-of-fit (Table 1).

### Responses of geographical assemblages of mosquito species

**Figure 4:**
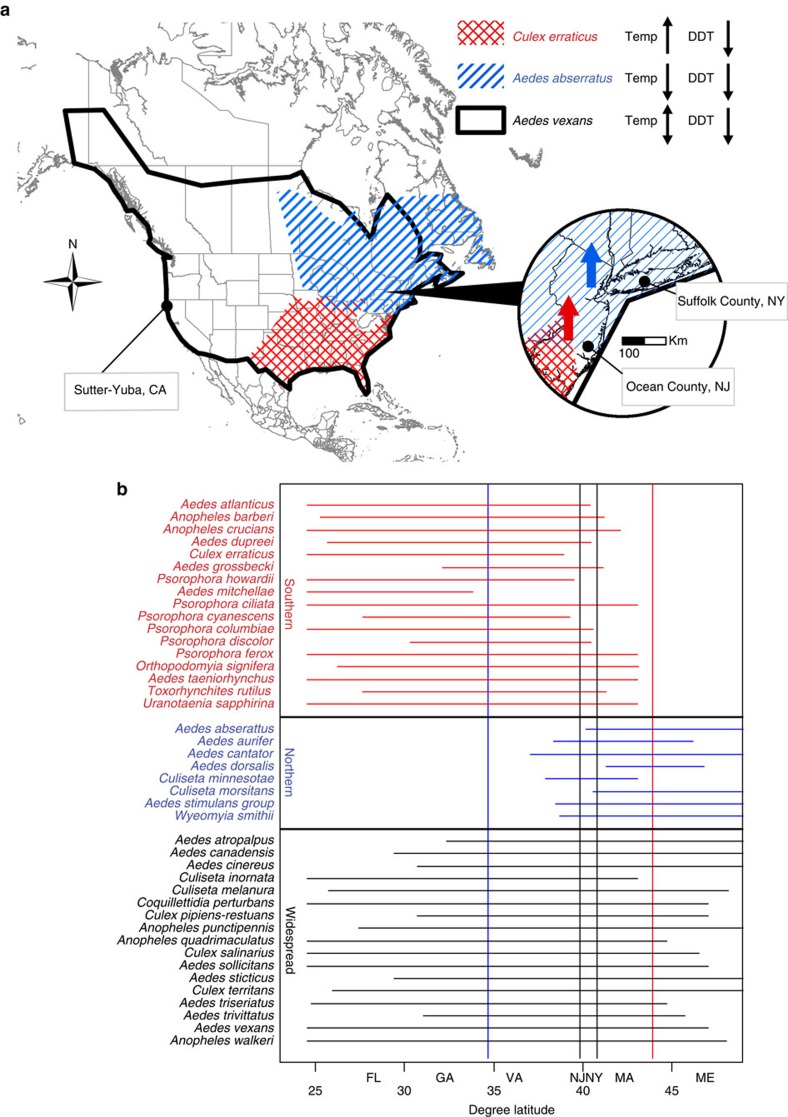
Predicted changes in the geographic distributions and abundance of mosquitoes. (**a**) Colours on the map show distributions for representative Southern (*Culex erraticus*), northern (*Aedes abserratus*) and widespread (*Aedes vexans*) mosquito species and the three study areas. Black arrows indicate predicted changes (up or down) in the species richness and abundance of these groups of mosquitoes if changes were primarily driven by temperature (Temp) or DDT. In the inset map, red (*Culex erraticus*) and blue (*Aedes abserratus*) arrows show the expected shifts in these species populations with increased temperature relative to the study sites. (**b**) The geographic ranges of mosquito species based on Darsie and Ward^39^ relative to geographic latitude and states, with the northern boundary of southern species given by the red vertical line and the southern boundary of northern species shown by the black line.

**Figure 5:**
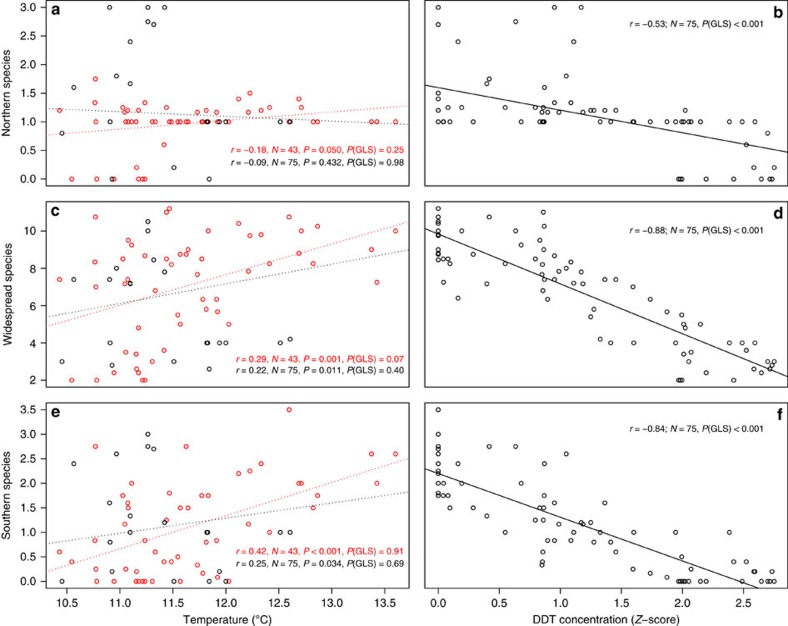
Relationships between mosquito species richness and temperature or DDT. The average number of mosquito species per trap are plotted against the yearly average temperature (in degrees Celsius; points in black are 1938–1959, before and during DDT use; points in red are 1960–2012 when DDT concentrations in the environment were declining) for Northern (**a**), Widespread (**c**) and Southern (**e**) mosquito species and plotted against DDT concentration (**b**,**d**,**f**, *Z*-score) in Suffolk County, New York. For the left panels, simple Pearson product-moment correlation coefficients, sample size, and *P*-values are given, followed by the *P*-value from GLS analyses that accounts for the residual temporal autocorrelation (‘*P*(GLS)'). Equations in red show analyses for just the red points (1960–2012), whereas equations in black show analyses for the full dataset 1938–2012. Dashed lines show the best fit lines but none were significant in the GLS analyses. In the right panels only the *P*-values from the GLS are shown (all Pearson product-moment correlation *P*-values for DDT were <0.001 for the full datasets and for the period 1960–2012).

**Figure 6:**
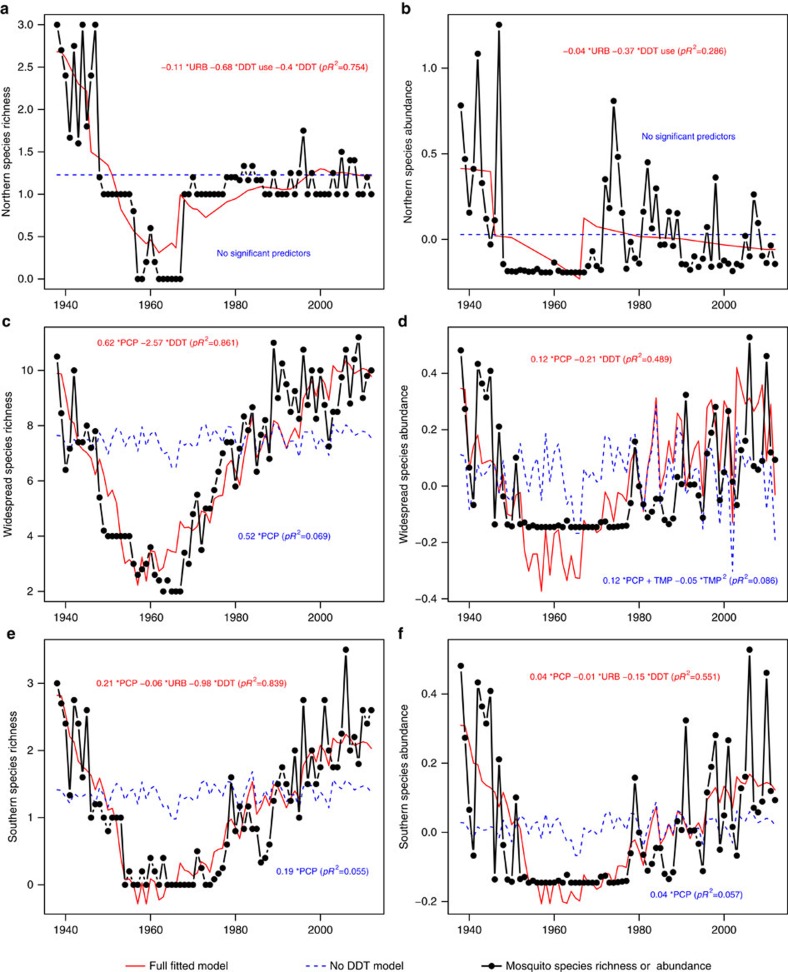
Regional fitted models and data for mosquito species richness and abundance in New York. Black line and dots show mosquito species richness for Northern (**a**), Widespread (**c**) and Southern (**e**) groups of mosquitoes or relative abundance (**b**,**d**,**f**). Red lines show full models, and blue dashed lines shows reduced models excluding DDT parameters. The equation in each panel gives the fitted model with all significant predictors (*P*<0.05) and fraction of variance explained (pseudo-*R*^2^, ‘*pR*^2^') from the generalized least squares models (red font-full model, blue font- reduced model with DDT parameters excluded). The abbreviations designate DDT concentration (DDT, *Z*-scores), DDT use by the mosquito control districts (yes/no), precipitation (PCP, standardized precipitation index), urbanization (URB, human population in 100,000), average annual temperature November through October (TMP, °C). For relative variable significance in the model see Table 1.

**Figure 7:**
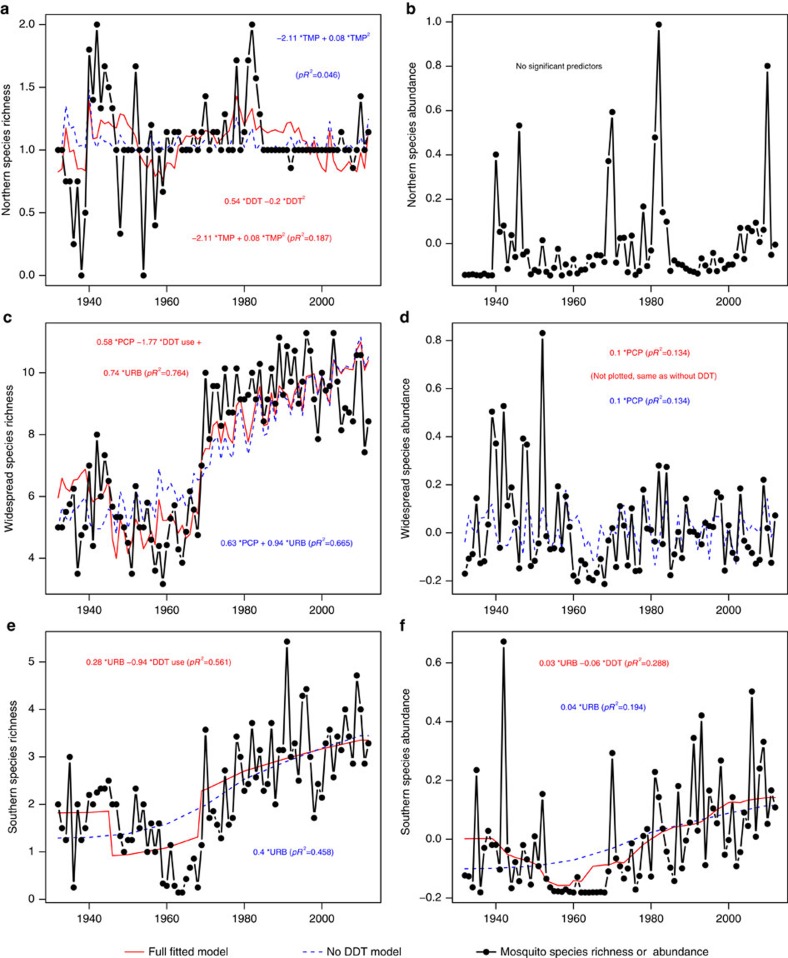
Regional fitted models and data for mosquito species richness and abundance in New Jersey. Black line and dots show mosquito species richness for Northern (**a**), Widespread (**c**) and Southern (**e**) groups of mosquitoes or relative abundance (**b**,**d**,**f**). Red lines show full models, and blue dashed lines shows reduced models excluding DDT parameters. The equation in each panel gives the fitted model with all significant predictors (*P*<0.05) and fraction of variance explained (pseudo-*R*^2^, ‘*pR*^2^') from the generalized least squares models (red font-full model, blue font- reduced model with DDT parameters excluded). The abbreviations designate DDT concentration (DDT, *Z*-scores), DDT use by the mosquito control districts (yes/no), precipitation (PCP, standardized precipitation index), urbanization (URB, human population in 100,000), average annual temperature November through October (TMP, °C). For relative variable significance in the model see Table 2.

**Figure 8:**
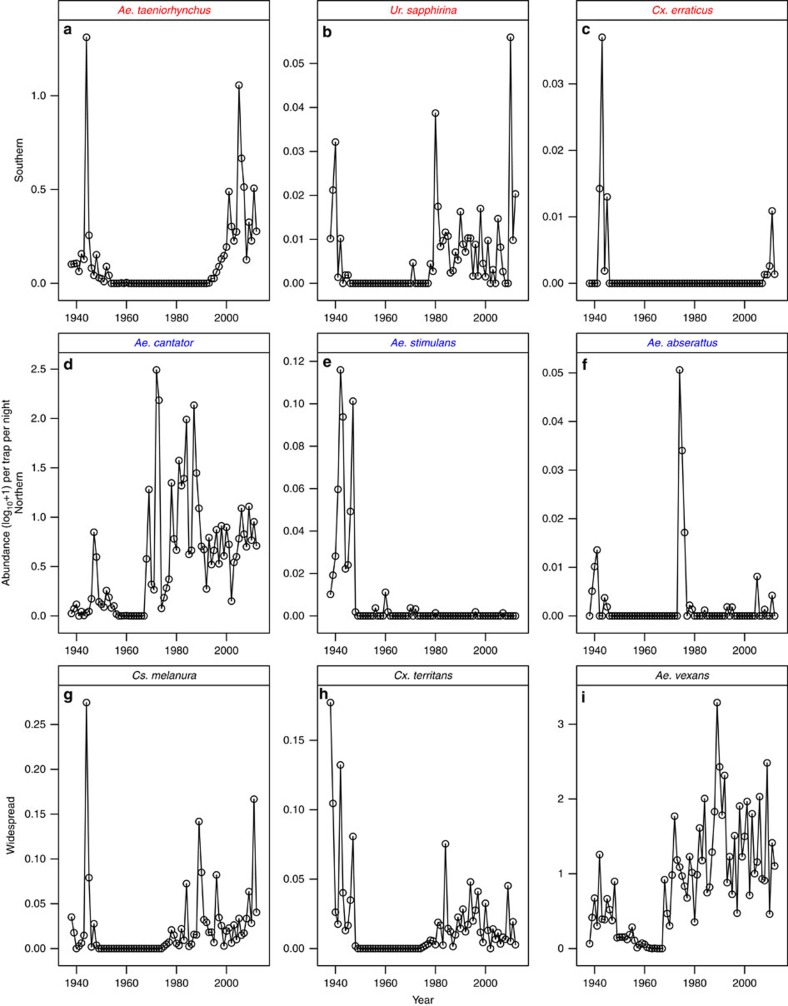
Abundance of representative mosquito species over time in New York. Panels show average number of female mosquitoes collected per trap per night. Southern species (panels **a**–**c**) with names in red. Northern species, (panels **d**–**f**) with names in blue. Widespread species (panels **g**–**i**) with names in black.

In our second set of analyses, we examined geographical subsets of mosquito species in the two study regions in the eastern USA (Fig. 4). The results were similar to the analyses on the full set of species examined together. Although some univariate correlational analyses were suggestive of temperature effects (Fig. 5), multivariate analyses that accounted for temporal autocorrelation in the residuals showed that DDT and urbanization were again the most important predictors for each of the three sets of mosquito species (Table 2; Figs 6 and 7), and temperature was non-significant. These analyses parallel trends of individual species in both relative abundance and presence/absence in individual traps in the study regions, which exhibited precipitous declines following commencement of DDT use in the late 1940s and variable degree of recovery after DDT use ceased in the late 1960s (Fig. 8).

## Discussion

There have been enormous changes in animal communities over the past century. Although many studies have found positive correlations between temperature and insect populations, most have been limited in temporal scope to the past five decades and nearly all of these studies have ignored the influence of land use or anthropogenic chemical use^1^,^3^,^5^,^6^,^7^,^16^. Our analyses, using invaluable long-term data collected by mosquito population monitoring programmes, showed that two other anthropogenic forces—DDT and land use—were the dominant drivers of mosquito populations and that recovery of populations and communities occurred as DDT concentrations in the environment waned. Patterns were remarkably strong given the substantial spatial heterogeneity present in mosquito populations, DDT use and urbanization. Surprisingly, we found little evidence that mosquito abundance or diversity responded to year-to-year variation or long-term warming trends in temperature, despite the presence of significant warming trends over time. Although simple univariate analyses with temperature sometimes produced significant correlations (Fig. 5), rigorous analyses that included other factors showed these correlations to be spurious. These results suggest that human alterations to animal communities can be pervasive, even if warming has had relatively little effect until now.

While our correlative analyses suggested that DDT was the strongest driver of mosquito populations overall, other factors, such as land use, that have changed monotonically over the last century, were also important in explaining patterns of change in mosquito communities. Human population growth and resulting urbanization, which is especially pervasive in the northeast USA but is occurring worldwide, was correlated with increased mosquito species richness and decreased relative abundance. Urbanization results in increased impermeable surfaces (for example, pavement) associated with buildings and roads, and decreases in forest cover, wetlands and other natural habitats. These changes alter mosquito larval habitats and populations of the hosts they feed on, and could also increase the permeability of the landscape for urban and anthropophilic mosquitoes and decrease movement for species more dependent on natural habitats^27^,^28^,^29^. The increase in species richness with urbanization likely reflects expansion of habitat for mosquito species associated with suburban environments and man-made wetlands^29^. The decline in abundance, which was especially apparent in abundant floodwater species, likely resulted from a reduction in natural wetland larval habitat (mainly salt and brackish marshes in the east, and freshwater habitats in the west) and increased light from growing urbanization which might reduce the attractiveness of the light-baited traps^22^. Urbanization, driven by human population growth and movement, has been a major driver of environmental change during the last century and is projected to increase substantially in the future across the globe^30^. Our results suggest that urbanization is likely to drive additional changes in mosquito communities, including the expansion of habitat for urban mosquitoes.

Although the effects of DDT on mosquitoes were evident in all three datasets, the quantitative impact varied among regions, likely due to different amounts of DDT used in these regions, the geographical context of the study areas, and the lengths of the time series. The amount of DDT used in the study area in NY was 1–2 orders of magnitude higher than that in NJ or CA^31^, which is consistent with the larger impacts of DDT on NY mosquitoes. The slower recovery of mosquito populations in the NY study area may have been partly due to lower connectivity to remnant populations due to being more geographically isolated on Long Island from many species' primary distributions to the south (Figs 4 and 8). Finally, the California dataset was the shortest in duration and, importantly, did not include the first 7 years of DDT applications, and this data gap may have resulted in the weak (and possibly spurious) temperature correlations detected in California mosquito populations.

Other studies on the long-term effects of DDT on insect communities and their ecosystem consequences are few, but also support the assertion that DDT has had widespread lasting effects. Long-term monitoring of moth populations in the UK initiated in 1933 detected significant declines of moth abundance and diversity through the 1950s during the period of peak DDT usage (cited in Fox^32^). In Canada, beetles declined in the diets of chimney swifts (*Chaetura pelagica*) closely following the increase in DDT concentrations in the environment from the 1940s through the 1960s^33^. Finally, there were unexplained significant increases in six species of insectivorous bats in the northeastern US from the 1980s until 2007 when white-nose syndrome, an emerging fungal disease, began to reverse these increases^34^. Our results demonstrating long-term effects of DDT on insect abundance offer a potential explanation for these patterns, and underscore the long-term influence of DDT on insects and their consumers. Other mosquito control activities also impact mosquito populations, and variation in these activities may be responsible for some of the unexplained variation we observed.

The widespread, long-term and lasting impacts of DDT and other organochlorine pesticides affected ecosystems worldwide in both temperate and tropical areas, as well as the oceans and the atmosphere^15^. Impacts of DDT likely played a role in historical trends in abundance, diversity and distribution of many animal species and may have been similar in spatial extent to those predicted to result from climate change. With the cessation of widespread DDT use for agriculture in most of the world, climate, land use changes and species introductions are expected to become the driving forces in altering mosquito populations. These anthropogenic processes have already facilitated the invasion and transmission of vector-borne pathogens such as West Nile, Zika, dengue, and chikungunya viruses^8^,^35^.

## Methods

### Study area

Mosquito data were collected in Suffolk County, New York, US (NY: 40.8° N, 73° W), Ocean County, New Jersey, US (NJ: 39.9° N, 74.2° W), and Sutter and Yuba Counties, California, US (CA: 39° 8′ 5″ N, 121° 37′ 34″ W) (Fig. 3). Suffolk (NY) and Ocean (NJ) counties are located along the Atlantic seaboard in northeastern US and are primarily suburban with a mix of residential, protected natural and limited agricultural areas. The landscape is a coastal plain topography with low elevation and numerous freshwater and tidal saltwater or brackish wetlands. Historically, both counties experienced fast population growth starting in the late 1940s, changing from mostly rural to mostly suburban. The current population of Suffolk County (NY) is 1.49 million with density of 632 residents per km^2^, whereas Ocean County (NJ) has a population of 0.58 million with density of 350 residents per km^2^. The Sutter and Yuba counties in California are located in the extensively farmed Central Valley. The landscape consists of urban core and outlying agricultural lands interspersed with rice fields, orchards, freshwater wetlands and protected natural areas. Historically, the Sutter-Yuba area underwent rapid conversion to agriculture in the early 20th century with the accompanying draining or reclamation of wetlands. The current population of the area is ∼165,000 people with density of ∼50 residents per km^2^.

### Mosquito data sources and collection

Mosquito surveillance was carried out by county mosquito control programmes since the 1930s (NY and NJ) or 1950s (CA). Permanent mosquito monitoring sites were established in the proximity of habitats producing biting mosquitoes. In NY and NJ, those habitats included saltwater or brackish tidal wetlands and the adjacent forested areas containing freshwater wetlands. These areas were representative of the mid-Atlantic coastal habitat, which experienced rapid urbanization in the 20th century. Despite significant suburban developments within the counties, much of the immediate natural areas near the traps have remained relatively unchanged since the programmes' commencement. Urbanization of the broader landscape during the study period (1932–2012) included conversion of agricultural and forested areas into mostly single family housing with dense road networks and associated commercial districts. Hence, the trap sites remained representative of the landscape changes that have occurred over much of the eastern Atlantic coast of the US. In CA, the trap sites were established in low and medium-density developed areas surrounded by large-scale agriculture representative of the Central Valley of California.

Adult mosquitoes were collected by New Jersey type light traps which consist of a 25 watt incandescent light bulb as the mosquito attractant, and a fan to draw the mosquitoes into a collection jar containing an insecticide for specimen knockdown. The original trap design and sampling protocols have been relatively unchanged since the 1930s^22^. The traps were deployed during the mosquito season (April–October) and operated every night. Specimens were retrieved 1–3 times per week, brought to the laboratory, and identified by trained entomologists under a microscope using published morphological keys. Only female mosquitoes were included in the analyses.

Mosquito trap sites were selected for the study based on their continuous record for the two locations starting in the 1930s. In NY, a total of 12 trap sites in five geographically proximate clusters were used for the analyses. Annual summaries were obtained from published reports and Suffolk County Vector Control records for the years 1938–2012 (with 1939 and 1975–76 missing). In NJ, a total of seven permanent trap sites were used in the analyses with the annual summaries for the years 1932–2012 obtained from the Ocean County Mosquito Extermination Commission. In CA, a total of four permanent trap sites were used in the analysis with the annual summaries obtained for the years 1954–2006.

### Mosquito data processing

We standardized the trap collections by trapping effort—the total number of females of each mosquito species caught in each trap was divided by the number of nights the trap was operated. Trapping effort was relatively consistent from year to year. If the trapping effort data were missing, the number of nights was interpolated from the previous and the following year. To avoid issues of spatial autocorrelation in abundance, all trap sites within each region were averaged to produce a single value expressed as an average number per trap per night for each species annually.

Mosquito species richness was calculated as the average number of species collected per trap per night. Average mosquito relative abundance was produced by standardizing or Z-transforming (that is, subtracting the mean and dividing by the standard deviation) the counts of females per trap night for each species over the entire study period. The *Z*-scores for individual species were then averaged by year in each study area. For illustration in the figures the *Z*-scores were re-scaled so that the minimum value was zero. Two recently introduced exotic species, *Aedes japonicus* (the Asian bush mosquito) and *Aedes albopictus* (the Asian tiger mosquito) were excluded from the analyses because their initial appearance in mosquito communities was unrelated to climate, land use or DDT. Morphologically indistinguishable species with similar geographic ranges were combined together: *Culex pipiens* and *Culex restuans* as *Culex pipiens-restuans*; *Aedes stimulans*, *Aedes excrucians* and *Aedes fitchii* as *Aedes stimulans* group; and *Anopheles crucians* and *Anopheles bradleyi* as *Anopheles crucians* group.

### Data sources and processing

Historical monthly temperature and precipitation data were obtained from the National Climatic Data Center for New York and New Jersey coastal divisions and California Sacramento Drainage division^36^ Several climatic variables were examined. Average annual temperatures for the current year were calculated between November of the preceding year and October of the current year to incorporate overwintering temperatures. Average seasonal temperatures including winter (December–February), spring (March–May), summer (June–August) and fall (September through November) time periods, and average temperatures of the coldest (January) and warmest (July) months were also included in the analysis. Minimum and maximum temperatures for all time periods were also calculated, but excluded from the analysis due to high correlation with average temperatures (*r*>0.9, data not shown). Finally, we calculated cooling degree days based on the day's average minus 18.3 °C (=65°F) and represent a heat index for a particular warm season of the year, that is, hotter seasons result in higher number of cooling degree days.

For precipitation, an annual average of the short term precipitation index (Standardized Precipitation Index, SP09) was used to capture precipitation patterns over time. A zero index value reflects the median of the distribution of precipitation, a −3 indicates a very extreme dry spell, and a +3 indicates a very extreme wet spell. The more the index value departs from zero, the drier or wetter an event lasting a given number (for example, nine for SP09) of months was when compared with the long-term climatology of the location. The index allows for comparison of precipitation observations at different locations with markedly different climates. For detailed description see Divisional Data Description, Standardized Precipitation Index (SP) section^36^. Total early season precipitation from January through April, and precipitation difference total (March+April)−total (January+February), which might be important for some mosquito species especially in California, were also examined in the analyses.

To examine the influence of DDT on mosquitoes, we included a binary yes/no indicator variable for periods when mosquito control agencies used DDT and a continuous variable representing the concentration of DDT in the environment, which reflects vastly larger amounts of DDT used in agriculture and forestry^14^. DDT was used for mosquito control in targeted applications within the study areas in 1946–1966 (Suffolk County, NY), in 1946–1968 (Ocean County, NJ) and 1946–1963 (Sutter-Yuba, CA). DDT or its derivatives have persisted in the environment and have been measured in sediment cores^37^. The amount and input of DDT into the environment was calculated based on dated sediment measurements from five lakes or bays in New York, New Jersey, and Connecticut, and California provided by the US Geological Survey^38^. The sediment data were available for 6–14 years dispersed between 1940 and 2004 depending on the sampling location. Missing annual DDT concentrations were linearly interpolated using na.approximate in the R package ‘zoo'. DDT concentrations before 1940, or after 2000, were assumed zero if missing. DDT concentrations were standardized (that is, rescaling to have a mean of zero and a standard deviation of one) for each of the sampling locations over the entire study period. The resulting *Z*-scores were then averaged by year to create an index of DDT amount deposited in the region where the study sites were located.

Historical census data for each county were obtained from the National Historical Geographic Information System (www.nhgis.org). The census was taken every 10 years from 1930 to 2010 and populations were interpolated to produce an annual estimate using na.approximate in R package ‘zoo'. The population in 2011–12 was assumed to be equal to that in 2010.

### Geographical grouping of mosquito species

The response of mosquitoes to temperature might differ depending on a species' distribution, and in the two study areas in eastern North America many mosquito species reach their northern or southern geographic limits, making it a useful area to examine range shifts^39^. In contrast, the distributions of almost all mosquito species in the study region in California spanned both north and south of the study area so differential responses to temperature based on geographical distributions were less likely. As a result, we only examined geographical groups of mosquitoes using the eastern USA datasets.

If temperature was the primary driver of changes in mosquito abundance and distribution in this region then higher temperatures would lead to increased abundance and richness of species with distributions to the south of the study areas as a warmer climate would facilitate their invasion, whereas more northern species should decrease in abundance or become extirpated from the study area as the region becomes too warm for them (Fig. 4, ref. 2). We used a recursive partitioning algorithm to divide the 42 mosquito species in both study regions into southern (17 species with northern range boundaries within 3.1° latitude of Suffolk County), northern (8 species with southern boundaries within 5.185° latitude of Ocean County) and widespread (17 other species) assemblages (Fig. 4; based on ref. 39) using the R package ‘rpart' v. 4.1-0 (ref. 40). The historical geographic ranges of mosquito species in North America were based on collection data reported through the 1970s^39^.

The classifications of two species were done manually to reflect details of their distribution. *Culiseta minnesotae* fit both southern and northern assemblages' criteria because of its narrow and patchy distribution around our study areas, however this species is generally classified as northern^39^ and was included as such in our analysis. *Culiseta inornata*, a mostly western species rare within our study area, was classified as southern by the software, but moved to the widespread species category based on a broad distribution from Yukon to Florida in North America.

### Statistical analyses

We used linear generalized least squares regression (gls, package ‘nlme') to analyze the time series of mosquito species richness and mosquito relative abundance. We accounted for the temporal autocorrelation in the time series residuals by specifying an autoregressive correlation structure. We used generalized least squares regression, rather than traditional time series approaches such as autoregressive or moving-average models that examine the change in time series, because the temporal resolution of our mosquito data was annual and as a result, the number of individual mosquitoes or mosquito species was more likely to be influenced by variation in the predictors in the current year than in past years. However, we also used wavelet analysis with Morlet wavelets (functions ‘analyze.wavelet' and ‘analyze.coherency' in the R package ‘WaveletComp' v.1.0) to examine the temporal scale or frequency of fluctuations in mosquito abundance and richness, as well as our predictors, and examined the coherence in fluctuations between time series.

Explanatory variables considered in model selection included temperature, precipitation, DDT amount index, DDT use over time (binary yes/no), human population (land use surrogate) and location (that is, County) as well as the interaction terms of each variable with location. The explanatory power of models was measured via a pseudo-*R*^2^, calculated as: pseudo-*R*^2^=1−[sum(model residuals)]^2^/[sum(null model residuals)]^2^, where the null model has an intercept and autoregressive terms or an intercept and random year effects only. We used the type=‘normalized' argument in *R* for extracting residuals for the generalized least squares (GLS) models that incorporates the autoregressive term, and un-normalized the residuals for both null and non-null models by multiplying by their standard deviation. Given the large number of temperature and precipitation variables, informative predictors were selected based on the reduction in Akaike Information Criterion (AIC) value (Table 1) using the stepwise selection procedure stepAIC in both directions^41^. Review of the resulting models indicated that uninformative parameters could be present in the AIC selection, a problem common in ecological modelling^42^. Further variable selection to obtain parsimonious models was performed using model-averaged parameter estimates (multi-model inference package ‘MuMIn' in *R*)^43^ with non-significant (*P*≥0.05) parameters dropped from the final model (Table 1). We checked for multicollinearity using variance inflation factors (VIF)^44^. Only multiple temperature variables had high VIF (that is, >10) in full models, and all temperature variables were usually dropped from models^44^. All final models had low VIF (that is, <3) suggesting lack of collinearity among the variables. All data were analyzed using R version 2.15.1 statistical software^45^.

### Data availability

All relevant data are available from the authors upon request.

## Additional information

**How to cite this article:** Rochlin, I. *et al*. Anthropogenic impacts on mosquito populations in North America over the past century. *Nat. Commun.* **7**, 13604 doi: 10.1038/ncomms13604 (2016).

**Publisher's note**: Springer Nature remains neutral with regard to jurisdictional claims in published maps and institutional affiliations.

## Figures and Tables

**Table 1:** Model comparisons and variation explained.

**Metrics**	**Species richness**			**Relative abundance**			**Variable description**
**State (location)**	**NY**	**NJ**	**CA**	**NY**	**NJ**	**CA**	
**Intercept only**	**(262.1)**	**(259.6)**	**(86.5)**	**(−12.3)**	**(78.1)**	**(132.3)**	
**Final model pseudo-*****R***^**2**^	**84% (115.1)**	**75% (143.2)**	**52% (45)**	**59% (−72.2)**	**15% (−95)**	**39% (108.6)**	
DDT amount	100% (29.2)		76% (13.3)	33% (19.4)	49% (4.9)	61% (4.3)	Standardized sediment cores
DDT use		16% (13.2)		5% (2.9)			For mosquito control
Urbanization		33% (20.9)	26% (2.9)	11% (7.7)		100% (7.9)	Population growth
Annual temp				5%* (0.9)			Average temperature
Spring temp			7% (4.3)				Average temperature
Summer temp							Average temperature
Fall temp						5% (4.2)	Average temperature
Winter temp							Average temperature
January temp							Average temperature
July temp							Average temperature
Cooling DD							Degree day average temperature −18.3 °C
PCP	4% (12.1)	0% (3.5)		9% (7.2)	46% (5.2)		Precipitation (index SP09)
PCP.Jan.Apr						7% (5)	Precipitation January to April
PCP.diff		2% (2.9)					Precipitation difference, [April+March]−[January+February]

The table shows the predictors examined in the analyses, the goodness-of-fit (pseudo-*R*^2^) value for the final model, and the Akaike Information Criterion (AIC) value in parentheses for an intercept-only model and the final model. Predictors retained in the final model for each dataset show per cent reduction in the goodness-of-fit of the final model (pseudo-*R*^2^) and increase in AIC in parentheses when removed, and may sum to more than 100% due to correlations among predictors. Higher values indicate predictors with higher contribution to the overall goodness-of-fit of the model. The goodness-of-fit of models was measured via a pseudo-*R*^2^, calculated as: *R*^2^=1−[sum(model residuals)]^2^/[sum(null model residuals)]^2^, where the null model has an intercept and autoregressive terms. Empty cells indicate non-significant variables that were not retained in the final model. The predictors include DDT amount (DDT, *Z*-scores), DDT use by the mosquito control districts (DDT use yes/no), urbanization (URB, human population, in 100,000), average annual (November through October), seasonal (spring, summer, fall, winter) or monthly (January or July) temperature (TMP, °C), cooling degree days (DD) based on the day's average temperature minus 18.3 °C, precipitation (PCP, standardized precipitation index SP09), total precipitation in January through April (PCP.Jan.Apr), precipitation difference [April+March]−[January+February] (PCP.diff).

^*^Main and quadratic terms.

**Table 2:** Variance explained of models for geographic assemblages of mosquitoes.

**Predictors**	**Southern**				**Northern**				**Widespread**			
	**Species richness**		**Relative abundance**		**Species richness**		**Relative abundance**		**Species richness**		**Relative abundance**	
	**NY**	**NJ**	**NY**	**NJ**	**NY**	**NJ**	**NY**	NJ*	**NY**	**NJ**	**NY**	**NJ**
DDT	100%		97%	32%	95%	76%†			92%		82%	
DDT use		18%			16%		90%			13%		
URB	7%	30%	18%	24%	65%		82%			48%		
PCP	2%		5%						4%	>1%	17%	100%
TMP						33%†					10%†	
*R*^2^, full model	0.839	0.561	0.551	0.288	0.754	0.187	0.286	NA	0.861	0.764	0.489	0.134

DDT, dichlorodiphenyltrichloroethane; NA, not available.

Best regression model goodness-of-fit and variable contributions are shown. The explanatory power of models was measured via a pseudo-*R*^2^, calculated as: *R*^2^=1−[sum(model residuals)]^2^/[sum(null model residuals)]^2^, where the null model has an intercept and autoregressive terms. Significant (*P*<0.05) variables retained in the final regression model were removed from the model singly and per cent reduction in the resulting pseudo-*R*^2^ compared with that of the final model is shown in the table. Higher values indicate variables with higher contribution to the overall goodness-of-fit of the model. The abbreviations designate DDT amount (DDT, *Z*-scores), DDT use by the mosquito control districts (DDT use), precipitation (PCP, standardized precipitation index), urbanization (URB, human population, in 100,000) and either an average annual November through October (widespread) or January temperature (northern) (TMP, °C). For regression coefficients see Figs 6 and 7.

^*^No significant predictors.

^†^Significant main and quadratic terms.
